# AI-assisted image analysis of *in vitro* oral cell morphology for early disease detection

**DOI:** 10.6026/973206300222817

**Published:** 2026-05-31

**Authors:** Nidhi Hirani, Ketankumar Jayantilal Prajapati, Ankita Shrivastava, Aditya S Dupare, Nilesh Dinesh Pardhe, Rakashree Chakraborty

**Affiliations:** 1 Department of Oral medicine and Radiology, Government Dental College and Hospital, Jamnagar, Gujarat, India; 2 Department of Oral and Maxillofacial Pathology, Siddhpur Dental College and Hospital, Siddhpur, India; 3 Department of Dentistry, Gajra Raja Medical College, Gwalior, Madhya Pradesh, India; 4 Department of Oral Medicine diagnosis & Radiology, Yogita Dental College & Hospital, Khed, Dist Ratnagiri, Maharashtra, India; 5 Department of Oral Pathology, ESIC Dental College & Hospital, Kalaburagi, Karnataka, India; 6 Department of Oral Medicine and Radiology, Maharishi Markandeshwar College of Dental Sciences and Research, Mullana, Ambala, Haryana, India

**Keywords:** Artificial intelligence (AI), oral epithelial cells, cell morphology, image analysis, early disease detection

## Abstract

Premalignant and malignant oral lesions pose a significant global health burden, with delayed diagnosis adversely affecting patient outcomes. Therefore, it is of interest to evaluate the diagnostic performance and reliability of an artificial intelligence (AI)-based image analysis system in identifying early morphological changes in cultured oral epithelial cells under simulated pathological conditions. Human oral keratinocytes were exposed to oxidative stress and inflammatory cytokines and phase-contrast images were analyzed using a convolutional neural network trained to classify normal, stressed and dysplastic cells. The AI system demonstrated high accuracy (94.2%), sensitivity (93.8%), specificity (95.1%) and strong agreement with expert cytopathologists (κ = 0.91), with reduced analysis time compared to manual evaluation. AI-based image analysis shows promise as a reliable adjunctive tool for early detection and screening of oral lesions.

## Background:

Oral diseases represent a wide range of pathology, including inflammatory diseases and possible malign lesions to blatant malignancies and impact billions of people worldwide [1]. The number of new cases and deaths annually that are caused by oral squamous cell carcinoma alone is about 377,000 and 177,000 respectively, which is one of the most frequent head and neck malignancies [2]. Although the treatment modalities have made significant progress, the oral cancer five-year survival rate has been a terrible disappointment at around 5065 mainly due to the fact that most patients are diagnosed at late stages when there are few treatment modalities as well as the fact that oral cancer patients have a low prognosis [3]. There is a consistent demonstration of early oral pathology detection, especially its detection at the premalignant or early dysplastic stage, having a profound effect on patient survival and treatment morbidity [4]. Tissue biopsy with subsequent histopathological analysis by a qualified pathologist is the gold standard of diagnosis of the lesions of the oral mucosa [5]. Nevertheless, this solution has a number of limitations, which are well documented. Oral epithelial dysplasia histopathological grading is infamously subjective and inter-observer and intra-observer variance has been reported to be up to 50% in certain studies [6]. Moreover, the procedure is labor intensive, specialized infrastructure is required and there is an ultimate delay between the acquisition of tissue and diagnostic reporting. Such limitations are especially troublesome in low-resource conditions and when conducting population-wide screening campaigns where high throughput and a high turnaround rate are needed [7]. Minimally invasive methods of sampling oral epithelial cells have also surfaced as cytological methods, such as oral brush biopsy and exfoliative cytology. The techniques enable the harvesting of parabasal and superficial cells without any form of surgical procedures hence making them appealing in screening [8]. However, traditional cytological interpretation has much in common with all the subjectivity and reproducibility problems of histopathological evaluation and its capability to identify early dysplastic alterations has been deemed to be inadequate to employ diagnostic purposes on its own [9]. The swift development of artificial intelligence, especially, the deep learning-based techniques that use convolutional neural networks, has provided revolutionary features in medical image analysis in various fields [10]. Pathology and cytology AI-based systems have been incredibly successful in classifying cervical cytology specimens, identifying malignant cells in pleural effusions and grading histological sections of different types of tumors [11]. The usage of deep learning as an analysis method of oral pathology images has been a topic of growing interest over the last few years. Research has shown that AI systems can identify oral histopathological images with as much or better accuracy as trained pathologists [12]. Moreover, there is a study about the possibility of using machine learning in the analysis of clinic photographs of oral lesions to facilitate risk stratification and triage [13]. Regardless of such positive results, there is a significant research gap associated with the systematic use of AI-based image analysis exploring the *in vitro* oral cell models to detect an early disease. The *in vitro* systems have unique benefits that can be applied to design and test AI diagnostic tools, such as the capability to accurately control and manipulate cellular niches, induce standardized and repeatable morphological changes and generate large annotated datasets that are needed to train powerful algorithms [14]. Few studies have examined the reliability of AI in differentiating between normal and pathologically altered oral epithelial cells in culture and a smaller number have used controlled *in vitro* models that model the progressive changes in morphology of early oral disease [15]. Some of the morphological parameters are nuclear-to-cytoplasmic ratio, nuclear pleomorphism, chromatin texture and cell boundary irregularity: all these parameters are established cytological parameters that pathologists use to identify dysplastic and malignant cells [16]. Computational image analysis as a quantitative measure of such properties can help minimize subjectivity and improve the reproducibility. Nevertheless, combining several morphological parameters into one AI classification system specifically tailored to analyze oral cells has not been studied to the full extent [17]. Therefore, it is of interest to report the diagnostic accuracy, sensitivity, specificity and reliability of an artificial intelligence-assisted image analysis system for automated classification of normal, stress-induced and dysplastic oral epithelial cells in controlled *in vitro* culture models.

## Materials and Methods:

## Study design:

It is an *in vitro* diagnostic accuracy study that was an experiment carried out in the Cell Biology and Digital Pathology Laboratory during the period between January 2024 and September 2024.

## Experimental groups and cell culture:

Primary human oral keratinocytes (HOKs) were cultured in a commercial cell line (ScienCell Research Laboratories, Carlsbad, CA, USA) and propagated in oral keratinocyte medium with oral keratinocyte growth supplement, 100 U/mL penicillin and 100 ug/mL streptomycin at 37°C at a humid atmosphere with 5% CO2. All experiments were done using cells between passages 3 and 6.

## Three experimental groups were determined:

[1] Group A (Normal Control, n = 450 images): Cells grown in normal conditions and were not treated, the morphology of healthy oral epithelials.

[2] Group B (Oxidative Stress/Inflammatory Model, n = 450 images): Cells that underwent hydrogen peroxide (H2O2 ) plus 20 ng/mL tumor necrosis factor-alpha (TNF- α) and 10 ng/mL interleukin-6 (IL-6) were exposed to hydrogen peroxide (H2O2 ) in 48 hours to model

[3] Group C (Dysplastic Model, n=450 images): Cells were placed on 0.5 μg/mL benzo[a]pyrene 72 hours followed by chronic exposure of low-dose 50 μM H2O2 48 hours in the presence of transforming growth factor-beta 1 (TGF-β1) (5 ng/mL) to induce morphological phenotype of early dysplastic alterations.

## Image acquisition:

The images in phase-contrast and fluorescense were obtained with an inverted microscope (Olympus IX73, Olympus Corporation, Tokyo, Japan) with a high-resolution digital camera (Olympus DP74) and a 20X objective lens. Each well had at least 15 non-overlapping fields of view. To enable nuclear morphometry, the nuclear staining was done on the Diamidino-2-phenylindole (DAPI) at 10g/mL and 10 minutes. The resolution of all pictures was 2048 x 2048 pixels in the form of TIFF. There were 1,350 images obtained in each of the three groups.

## Pre-processing and feature extraction of image:

The preprocessing of the images was carried out under the standardized protocols that were implemented on Python 3.10 with the libraries OpenCV and scikit-image. Background subtraction, contrast enhancement with adaptive histogram equalization, reduction of Gaussian noise and automated cell segmentation with watershed algorithm and Otsu thresholding were used as preprocessing. After segmenting, the individual cell morphological features were obtained, such as nuclear area, cytoplasmic area, nuclear-to-cytoplasmic (N/C) ratio, nuclear circularity index, cell boundary irregularity index (fractal dimension), chromatin texture (entropy, contrast, homogeneity and correlation based on gray-level co-occurrence matrices) and cell elongation factor. Each segmented cell had 14 morphological parameters that were calculated.

## AI model architecture and training:

A convolutional neural network (CNN) with a custom structure was created with the help of TensorFlow 2.12 and Keras frameworks. It was an architecture comprising of five convolutional blocks, each block has two convolutional layers, each with 3 by 3 kernels, batch normalization, rectified linear unit (ReLU) activation and max-pooling layers. Conventional blocks were then succeeded by a global average pooling layer, two fully connected dense layers (512 and 256 neurons, respectively) with dropout regularization (dropout rate = 0.4) and a softmax output layer (three nodes each in the three classification categories normal, stressed and dysplastic). Stratified sampling was used to split the dataset (70% training, 15% validation and 15% testing) in order to have all three classes (proportional) in the three segments. The training set was subjected to the techniques of data augmentation, such as random rotation (15 degrees), horizontal and vertical flipping, brightness adjustment (10 percent) and elastic deformation. The model was trained on 150 epochs, the Adam optimizer with the initial learning rate of 0.0001 and a batch size of 32 and categorical cross-entropy loss function. Early stopping and patience of 20 epochs was used to avoid overfitting.

## Expert human assessment:

Three boards's certified oral and maxillofacial cytopathologists with at least 10 years of diagnostic experience classified the same test-set of 202 images (single images) independently as either normal, stressed, or dysplastic. The pathologists were not informed of the experimental group assignment and also of the assessment of their colleagues. Majority (agreement of at least 2 of 3 concordant diagnoses) was used to determine consensus classification.

## Statistical analysis:

The AI model diagnostic accuracy measures, which are sensitivity, specificity, positive predictive value (PPV), negative predictive value (NPV) and the overall accuracy were estimated using the expert consensus classification as the reference standard. The kappa coefficient determined by Cohen was calculated to identify the agreement between AI and expert classifications and also, inter-observer agreement between the three pathologists. ROC curves were developed and each category of classification was provided with the area under the curve (AUC) based on one-versus-rest methodology. One-way analysis of variance (ANOVA) and Tukey post-hoc test were used to compare m orphological parameters (normally distributed data) and Kruskal-Wallis test/Dunn post-hoc correction (non-normally distributed data) to conduct the comparison. The Mann-Whitney U test was used to compare the time of analysis of AI and human assessment. Statistical tests were done by using IBM SPSS Statistics 28.0 (IBM Corporation, Armonk, NY, USA) and Python scikit-learn library. The statistical significance was determined as p < 0.05.

## Results:

Quantitative morphological analysis revealed significant differences across all three experimental groups for the majority of measured parameters. Cells in the dysplastic model group exhibited the highest nuclear-to-cytoplasmic ratios, greatest nuclear irregularity and most heterogeneous chromatin texture. The stress-induced group demonstrated intermediate morphological changes between normal and dysplastic phenotypes. Detailed morphological parameters are presented in Table 1. The CNN model achieved an overall classification accuracy of 94.2% (190/202 images correctly classified) on the held-out test set. Class-specific performance metrics are presented in Table 2. The model demonstrated highest accuracy for normal cell classification and lowest, through still robust, accuracy for the stressed phenotype category, likely due to the transitional nature of stress-induced morphological features. The area under the ROC curve was 0.98 for normal, 0.95 for stressed and 0.97 for dysplastic classification categories. The Cohen's kappa coefficient between AI classification and expert consensus was 0.91 (95% CI: 0.86-0.96), indicating almost perfect agreement. Inter-observer agreement among the three cytopathologists ranged from κ = 0.78 to κ = 0.84, with a mean of κ = 0.81, representing substantial agreement. Notably, the AI system's agreement with the consensus exceeded the pairwise agreement among individual experts. The mean processing time for the AI system was significantly shorter than manual expert assessment. Comparative metrics are summarized in Table 3. The confusion matrix for the AI model revealed that the most common misclassification pattern involved stressed cells being classified as dysplastic (5 of 12 errors) or normal cells being classified as stressed (4 of 12 errors), consistent with the morphological continuum between these categories.

## Discussion:

The current experiment shows that the system of image analysis based on the convolutional neural network is able to effectively classify *in vitro* cultured human oral keratinocytes with normal, stress-induced and dysplastic morphology phenotypes with an overall accuracy of 94.2 percent and near flawless consensus with the cytopathologist expert. These results have significant implications in the future creation of AI-based tools to screen early oral diseases. The morphological parameters obtained in this research have a close relationship with the existing cytological parameters in the clinical oral pathology. The gradual type of morphological changes of our *in vitro* model i.e., the gradual rise in nuclear-cytoplasmic ratio of normal to stressed cells to dysplastic cells are reflected in the morphological changes that have been extensively documented in the clinical samples of oral epithelial dysplasia [18]. We find our N/C ratio of 0.47 with the 0.47 value of moderate-severe dysplasia in clinical study of oral cytology [19]. Likewise, the reductions in nuclear circularity as well as the rise in chromatin texture heterogeneity indicate the nuclear pleomorphism and hyperchromasia, which are the characteristic features of the dysplastic transformation in the oral epithelium [20]. The performance of our CNN model with an accuracy of 94.2% in classification is equal to or better than the already reported AI performance in oral pathology applications. Deep learning based studies on histopathological classification of oral squamous cell carcinoma have achieved an accuracy of between 87% and 97% [21]. Nevertheless, it cannot be directly compared to these studies since on average they made use of tissue level histopathological images as opposed to cell level morphological analysis as was conducted in the current study. Machine learning applied in oral cytology images has been shown to provide classification accuracy ranging between 85 and 96 percent in identifying normal and malignant cells and this finding is generally consistent with our results [22]. One of the most interesting findings is that the agreement of the AI system with the expert consensus (0.91) was higher than any individual pathologist with the expert consensus (0.78-0.84). This fact is aligned with the emerging evidence of literature that shows that AI systems can equal or surpass the performance of individual experts in tasks of pattern recognition and be more consistent [23]. The inter-observer agreement among pathologists in our study with kappa values in the significant but imperfect category supports the long- term issues of the reproducibility of the subjective morphological evaluation in oral pathology [24]. The ability of AI to offer standardized and repeatable tests is one of its strongest benefits in the diagnostic process. The difference in processing time between the AI and human evaluation, around 1.8 seconds in comparison with 40-50 seconds per image, is dramatic to emphasise the possibilities of the AI-assisted analysis in the screening of high-throughput features. It may lead to significant changes in the diagnostic turnaround time and resource usage to the extent that it can be extrapolated to large-scale screenings with thousands of samples to imply this efficiency gain [25]. Moreover, the availability and the speed of the AI system on a regular basis indicate that it could be used in point-of-care facilities and telemedicine, where a quick initial evaluation would be clinically beneficial [26]. There are both the benefits and drawbacks of the usage of controlled *in vitro* models in this study. It was enabled by the capability to accurately control cellular environments and produce standardized morphological phenotypes to create a well-defined, annotated training dataset, which is essential in building sound AI classifiers [27]. The model of oxidative stress and inflammatory cytokines applied to Group B cells and recapitulated the main features of the tissue microenvironment that was studied in oral lichen planus and chronic periodontitis, which are considered risk factors of malignant transformation [28]. The dysplastic model of carcinogen induced in Group C cells gives rise to morphological changes which are reminiscent of early oral epithelial dysplasia, which include emphasized nuclear size, distorted N/C ratio and disorganized chromatin pattern [29]. Yet, significant shortcomings should be admitted. Monolayer cultures *in vitro* do not completely reproduce the three-dimensional tissue structure, cell-cell interactions and micro environmental effects of the body, which is only achievable *in vivo*. Although the morphological properties of cultured cells display structural similarities with their *in vivo* counterparts, they might vary in the subtle details that might influence the generalizability of AI classifiers training on *in vitro* measurements only [30]. Also, the three category classification system that was used in this research is a simplified version of the morphological continuum that is seen in the progression of oral diseases. This would have to be used clinically with the capacity to differentiate between a wider spectrum of pathological conditions such as different grades of dysplasia and other benign reactive processes capable of simulating dysplastic changes [31]. The patterns of misclassification that are evident in this study can give valuable insights on how the models can be refined in the future. The existence of errors in the boundary between the stressed and dysplastic categories is a sign of the true biological overlap of the inflammation- related changes of cells and the initial cases of dysplasia, a fact that daunts the human pathologists too [32]. The above observation indicates that one potential improvement of the AI system in the future is the inclusion of more information of the biomarkers, including immunocytochemical indicators of proliferation or tumor suppressor genes expression, to enhance discrimination at these diagnostic cutoffs [33].

Combining quantitative morphometric data and deep learning-based image classification is an orthogonal method that exploits the advantages of both the feature engineering and feature extraction feature. The past studies have shown that mixed methods incorporating handcrafted morphological features and CNN-based features may be better than each in cytological classification tasks [24]. Both styles were present in our model architecture, with the 14 predefined morphological parameters extracted and the automated feature learning of the CNN, which may be one of the reasons of the good overall result. The next phase of AI system validation should be conducted by using clinical oral cytology specimens to determine its translational potential. Multi-centered studies that will involve a wide range of patients, different conditions of image capture and a much wider range of category of oral pathology will be necessary in determining clinical utility [35]. Also, the creation of explainable AI methods that can emphasize the morphological characteristics precisely in the classification decision would further increase clinical adoption and integration into the established diagnostic practices [36].

## Conclusion:

The convolutional neural network-based artificial intelligence system showed high accuracy and reliability in classifying *in vitro* cultured human oral keratinocytes into normal, stress-induced and dysplastic phenotypes. It achieved excellent diagnostic performance with rapid analysis time, indicating strong potential for objective, reproducible and high-throughput screening of early oral cellular changes. Although further clinical validation is required, these findings suggest that AI-assisted morphological a nalysis may become a valuable adjunct for early detection of oral diseases.

## Figures and Tables

**Table 1:** Quantitative morphological parameters across experimental groups (Mean ± SD)

**Parameter**	**Group A (Normal)**	**Group B (Stressed)**	**Group C (Dysplastic)**	**p-value**
Nuclear Area (μm)^2^	78.4 ± 12.3	98.7 ± 18.5	134.2 ± 26.8	<0.001*
Cytoplasmic Area (μm)^2^	412.6 ± 58.9	356.3 ± 67.2	289.4 ± 72.1	<0.001*
N/C Ratio	0.19 ± 0.04	0.28 ± 0.06	0.47 ± 0.11	<0.001*
Nuclear Circularity Index	0.91 ± 0.04	0.82 ± 0.07	0.68 ± 0.12	<0.001*
Boundary Irregularity Index	1.04 ± 0.03	1.12 ± 0.06	1.28 ± 0.09	<0.001*
Chromatin Entropy	4.21 ± 0.38	5.14 ± 0.52	6.73 ± 0.67	<0.001*
Chromatin Contrast	12.4 ± 3.2	18.7 ± 4.8	28.9 ± 6.5	<0.001*
Cell Elongation Factor	1.18 ± 0.11	1.34 ± 0.16	1.62 ± 0.24	<0.001*
*One-way ANOVA; all pairwise
comparisons significant at
p < 0.05 by Tukey's
post-hoc test.

**Table 2:** AI model diagnostic performance by classification category

**Metric**	**Normal(Group A)**	**Stressed (Group B)**	**Dysplastic (Group C)**	**Overall**
Sensitivity (%)	95.5	91.2	93.8	93.5
Specificity (%)	97	94.8	95.1	95.6
Positive Predictive Value (%)	94.1	89.7	92.6	92.1
Negative Predictive Value (%)	97.6	95.7	95.8	96.4
Accuracy (%)	96.5	93.6	94.6	94.2
AUC (ROC)	0.98	0.95	0.97	0.97
F1 Score	0.948	0.904	0.932	0.928

**Table 3:** Agreement and processing time comparison between AI and expert assessment

**Parameter**	**AI System**	**Expert 1**	**Expert 2**	**Expert 3**	**p-value**
Accuracy vs. Consensus (%)	94.2	90.6	88.1	91.6	-
Kappa vs. Consensus	0.91	0.84	0.78	0.84	-
Mean Analysis Time per Image (seconds)	1.8 ± 0.3	42.5 ± 11.2	51.3 ± 14.7	38.9 ± 9.8	<0.001†
Total Test Set Analysis Time (minutes)	6.1	143	172.7	130.9	<0.001†
Misclassified Images (n)	12	19	24	17	-
†Mann-Whitney U test
comparing AI versus pooled expert analysis time.
